# First Reported CNS Infection with *Mycobacterium xenopi*—Illustrative Case Report and Therapeutic Protocol Suggestion

**DOI:** 10.3390/reports9020099

**Published:** 2026-03-26

**Authors:** Leonidas Trakolis, Simeon Metallidis, Athanasios K. Petridis

**Affiliations:** 1 Department of Neurosurgery, Agios Loukas Clinic Thessaloniki, 55236 Thessaloniki, Greece; 2 Infectious Diseases Unit, 1st Internal Medicine Department, AHEPA University Hospital, 54636 Thessaloniki, Greece; 3 Medical School, Heinrich Heine University Duesseldorf, 40225 Duesseldorf, Germany

**Keywords:** CNS infection, *Mycobacterium xenopi*, dural infection

## Abstract

**Background and Clinical Significance:** Mycobacterial infections of the central nervous system (CNS) are a serious condition that demands a rapid interdisciplinary approach according to the most recent protocols. Some cases, however, are unique and not yet reported in the literature. **Case Presentation:** We report such a case, a 42-year-old Greek male patient with a *Mycobacterium xenopi* infection of the cerebrodura near the cavernous sinus, an infection not yet reported in the literature and a real challenge for the treating doctors due to the location of the infection and the complication-rich course. **Conclusions:** This is the first reported intracerebral infection with *Mycobacterium xenopi*. We share our experience to assist clinicians with similar cases.

## 1. Introduction and Clinical Significance

Mycobacterial infections of the CNS are not uncommon, especially in developing countries. Although most cases are caused by *Mycobacterium tuberculosis* [1], further non-tuberculous mycobacterial infections have been mentioned in the literature [2]. The symptoms are very unspecific and often misleading. They can vary from meningismus, photophobia, nausea, vomiting to altered consciousness, while in more uncommon cases the patients suffer from cranial nerve paresis, seizures and/or hemiplegia [3]. The diagnosis is usually challenging [3], due to either the location of the infection or the fact that the (gold standard) Ziehl–Neelsen acid-fast bacilli (AFB) stain requires large amounts of CSF and has low sensitivity [4]. Most of the patients do not have systemic disease, so the blood examinations and cultures remain normal. On the other hand, PCR (polymerase chain reaction) detection of microbial DNA is notorious for false negative results due to the low bacterial load in CNS infections and incomplete removal of inhibitory substances [5,6]. An MRI of the brain would provide a clear picture of the problem but its specificity is low, leaving tumors, lesions or various kinds of infections as potential differential diagnoses [1]. There is no established treatment for nontuberculous mycobacterial CNS infections, so most clinicians use conventional antitubercular drugs, often resulting in treatment failure, high morbidity and mortality [7].

*Mycobacterium xenopi* was first isolated on frog skin in 1959 by Schwabacher and initially called *Xenopus laevis* [8]. The first human infections were reported in 1980 in a case series report from Costrini et al. [9]. After that, various reports have identified *M. xenopi* as an emerging bacterium for pulmonary diseases [9,10,11,12,13,14]. Interestingly, many cases are reported as nosocomial infections [15]. The pathogen is spread from the environment to humans and from human to human through aerosol inhalation or ingestion [16], although it has been isolated in tap water [17]. It affects mostly elderly or immunosuppressed patients (for example HIV patients) [15]. The first spinal infection with *M. xenopi* was over 30 years ago [18] and since then more reports have followed [19,20,21,22,23,24]. A recent review of *M. xenopi* spinal infections identified 14 cases with such an infection. A purified protein derivative (PPD) skin test was performed in seven cases, with only one positive result, highlighting how challenging the diagnosis of such infections can be. Eleven patients improved after antitubercular treatment [24]. However, in patients with pulmonary *M. xenopi* disease, the success of treatment is not so high. The mortality rate over two years can reach 54%, despite the antitubercular treatment [25]. Interestingly, no brain infection with *M. xenopi* has yet been reported in the literature.

Clinical Significance: We report such a case, a 42-year-old Greek male patient with a *Mycobacterium xenopi* infection of the cerebrodura in the region of the cavernous sinus, an infection not yet reported in the literature and a real challenge for the treating doctors due to the location of the infection, the difficulty in diagnosing it and the complication-rich course.

## 2. Case Presentation

**Figure 1:**
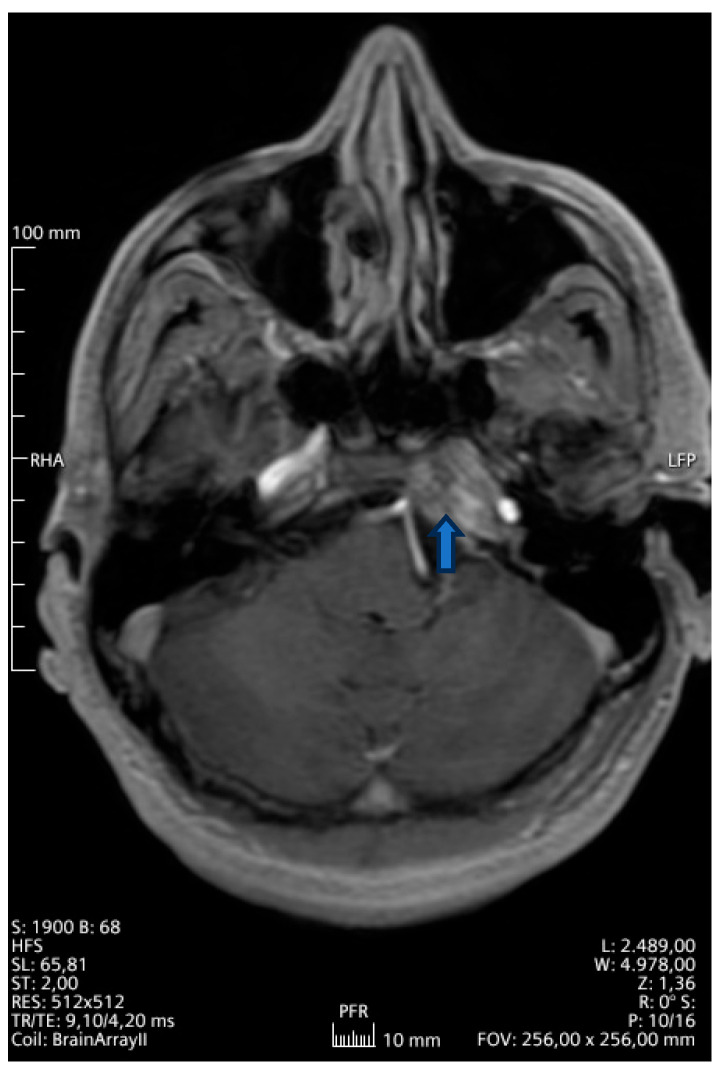
Initial MRI of the brain (T1 sequence gadolinium-enhanced) showing a lesion on the left cranial base (arrow) medially to the petrosal segment of the carotid and growing into the cavernous sinus.

**Figure 2:**
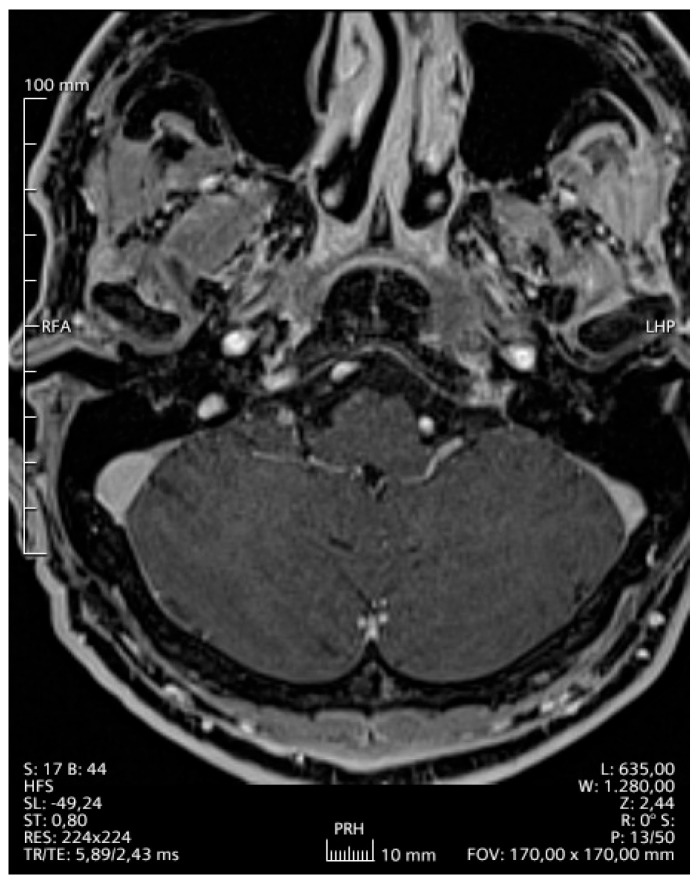
Second MRI 10 months after the first MRI and 8 months after initiation of the treatment against *Mycobacterium xenopi,* (T1 sequence gadolinium-enhanced) showing no gadolinium-enhancing lesions.

A 42-year-old Greek male patient presented with sialorrhoe, hypoglossal palsy and left sided abducent nerve palsy. The patient was otherwise healthy and was working in the marble industry. The magnetic resonance imaging (MRI) examination (Figure 1) showed a T2-hyperintense lesion of the sphenoid bone to the petrous ridge. Following an ear-nose-throat specialist (ENT) consultation, he started corticosteroid therapy (dexamethasone 3 × 12 mg), which led to a deterioration of the symptoms within three weeks. The urine culture was pathogen-negative, while negative was also the result of examinations for Bondia, Brucella and Coxiella infections. We suspected a *Mycobacterium tuberculosis* infection, but the sputum and blood culture were negative as well as the Mantoux test (or PPD test—purified protein derivative). The blood cultures were also negative for any pathogens; human immunodeficiency virus (HIV), hepatitis C virus (HCV) and hepatitis B virus (HBV) examination were likewise negative. Furthermore, rheumatological examination showed a slightly decreased hemopoetin value (1.9IU-lt, with normal values 2.6–3.4), although this could be due to the high-dose corticosteroid therapy. A summary of the most important blood and urine values can be found in Table 1. A computed tomography (CT) scan of the whole body showed no further pathology. An empiric therapy with vancomycin and ciprofloxacin combined with dexamethasone 2 mg twice every day was initiated. For four weeks we gradually increased the dexamethasone dose (3 × 8 mg) due to the further deterioration of the patient and the suspicion of an autoimmune disorder. The patient developed a more serious glossopharyngeal and abducens nerve palsy than before and was unable to eat or drink on his own. Consequently, we inserted a nasogastral tube for his nutrition and proceeded with an open biopsy of the dura matter, the cerebrospinal fluid (CSF) and adjacent structures of the cavernous sinus about two months after the first manifestation of the symptoms. The histological results showed atypical inflammatory cells in the dura matter and a negative CSF culture, supporting the theory of a meningioma. Due to these inconclusive results and the fact that intraoperatively there was no sign of a tumor, we performed a PCR test of the intraoperatively acquired samples (brain, dura matter and CSF), which revealed *Mycobacterium xenopi* DNA. The PCR was performed with a VisionArray MYCO chip 2.0 CE, IVD kit (ZytoVision GmbH, Bremerhaven, Germany), which can identify over 18 different mycobacteria (ITS region/IS6110Region). Due to the lack of similar primary CNS infections in the literature, we started an empirical triple antimycobacterial therapy with ethambutol 3 × 500 mg, rifampicin 2 × 300 mg and clarithromycin 2 × 500 mg, while we kept the corticosteroid therapy; the patient could be discharged in slightly better neurological condition and without the nasogastric tube. One month after initiation of this treatment, the patient presented to our emergency department with difficulty in breathing and cold legs. He was diagnosed with thromboembolism of the deep veins and the peripheral pulmonary arteries. Rifampicin was stopped and the patient received antiplatelet therapy, first heparin and then argatroban, due to slight thrombocytopenia and suspected HIT (heparin-induced thrombocytopenia). A month later, due to a deteriorating general condition, he was admitted once more to the emergency department of our clinic. MRI examination of the brain showed great improvement of the dural infection. However, a CT scan of the thorax showed an atypical infection of the lungs. The culture, cytological examination and PCR of a bronchoscopy-acquired sample confirmed an aspergillus infection and we added antimycotic medication to his therapy (voriconazole 2 × 200 mg). In addition, we gradually stopped the corticosteroid therapy, as the patient presented many side effects (depression, peripheral neuritis, skin thinning and diabetes). Thankfully, all of those side effects disappeared shortly after ceasing the therapy and the patient’s condition improved within a few days. The glossopharyngeal and abducens nerve palsies improved significantly and he could eat and swallow without difficulty. Six months after initiation of the antimycobacterial treatment, an MRI scan of the brain (Figure 2) was without any pathologic signs. We discontinued the antimycotic therapy after 6 months due to complete resolution of his lung infection and minor dermatological side effects. The antimycobacterial treatment was completed after 9 months. The patient is now healthy and started his daily routine and work.

## 3. Discussion

Nontuberculous CNS infections are rare and mostly seen in immunosuppressed patients, with HIV patients having a prevalence of 1.3% [2,26]. Therefore, there is no gold standard for diagnosis and therapy and the mortality rate is high [2,7]. The symptoms are in most cases typical of meningitis and biochemical examination of the blood and CSF can be useful. In our case, the patient presented with a rather rare symptomatology (cranial nerve palsies) for mycobacterial meningitis without meningismus, fever or photophobia, making the diagnosis more difficult. He was otherwise healthy, young and was working full time. Although males are most commonly affected by nontuberculous mycobacteria [1], the fact that he was otherwise healthy and immunocompetent is a rare coincidence. Furthermore, the location of the lesion in the skull base and next to the cavernous sinus made acquiring the specimen very difficult. Despite the possibility of finding such an infection in blood and CSF cultures, we could not identify it, while various additional tests for *M. tuberculosis* and HIV remained negative. Although corticosteroids are widely used in such infections [27,28], in our case they were ineffective and rather harmful for our patient, causing a variety of side effects. A corticosteroid therapy would be effective against an autoimmune disorder as well, although our test results were negative for the most common ones.

The source of the infection remains unclear. The patient reached us outside the hospital for the first 3 months before he was hospitalized, and had never had any surgery before the manifestation of this infection, so it could not be a nosocomial infection or due to hospital water. Furthermore, the region he lives in reported no further *M. xenopi* infections of any type. We concluded that it could be acquired at work due to his contact with stagnant water in the marble industry. MR imaging was rather unspecific, so we had to exclude autoimmune, neoplastic and rheumatological disorders. The location of the lesion near the cavernous sinus was very challenging for any kind of biopsy and, although we consulted our ENT colleagues, an endonasal biopsy was too dangerous while a transcortical approach through the left hemisphere could cause more neurological symptoms to the patient than he already had. The rapid deterioration of the symptoms, the lack of specific findings and the failure of primary therapy forced us to act in the least invasive way, approaching the lesion between the dural leaves. This gave us the chance to approach the lesion extra- and intradurally reaching the three trigeminal ganglions and the cavernous sinus without perforating the cortex, while acquiring various valuable specimens (dura matter, CSF and some cortex). The patient recovered rapidly from the surgery and could be discharged from hospital three days later without any new neurological deficiencies.

Another pitfall of the case was the first histological finding. Due to inflammatory and thickened dural cells, there was high suspicion of a meningioma, one of our differential diagnoses. Fortunately, during the surgery we had a clear view of the field and could exclude a meningioma, enabling us to further look for another cause. If the biopsy were performed in a minimally invasive way, the histological findings would be misleading and the patient would have received radiation therapy, with fatal consequences. Furthermore, since we could not identify anything pathological during surgery, the amount of the specimens taken was small and from macroscopically healthy tissue. This thwarted our ability to perform further tests (culture, antibiogram, etc.) to validate the diagnosis. The choice of therapy was straightforward due to the fact that the antimycobacterial therapy available is generally limited [2,7]. The duration of the therapy was decided according to the findings in the follow-up MRI examinations and the improving condition of the patient. Aspergillus-induced pneumonia is relatively common in immunocompromised patients and those under long corticosteroid treatment, and should always be considered in such cases [29]. In addition, rifampicin has been associated with venous thromboembolism events, deep vein thrombosis, pulmonary embolism and disseminated intravascular coagulation [30,31,32], further increasing the thrombosis risk in patients with tuberculosis. The exact mechanism is still unclear, but rifampicin is an effective liver enzyme inducer and affects cytochrome P450 [33,34,35].

This case taught us valuable lessons for the diagnosis and treatment of rare nontuberculous CNS infections. The diagnosis is challenging and should include not only standard procedures (blood and CSF cultures and biochemistry tests) but also molecular diagnostics and, when possible, biopsy of the lesion. In atypical lesions, always think of nontuberculous mycobacteria and perform a Ziehl–Neelsen acid-fast bacilli (AFB) stain of the blood, CSF and biopsy samples. Rapid deterioration of the symptoms means an active and aggressive disease that has to be treated. In some cases, risks have to be taken for the patient’s greater good. Last but not least, antimycobacterial medication and high-dose corticosteroid therapy may cause serious side effects and patients should be closely monitored.

## 4. Conclusions

This is the first reported primary mycobacterial infection of the cerebrodura with *M. xenopi* without any other focus, and should serve as orientation for future cases. If the exact diagnosis had failed (i.e., no PCR for atypical bacteria) the conclusion of the disease would be fatal. We introduce the diagnosis and treatment of such an atypical dural nontuberculous mycobacterial infection. During the treatment of the disease some complications occurred, which highlight the need for constant observation of the patient. The lung embolism and aspergillosis could have become fatal had they not been recognized in time, which led us to adapt the therapy regime by removing one antibiotic and adding an antimycotic agent later.

## Figures and Tables

**Table 1:** A summary of the most important blood and urine examinations performed.

	Initial Lab.	4 Months	Norm.
WBC	13.9 (10^3^/μL)	7.6 (10^3^/μL)	4.5–10.5 (10^3^/μL)
Neutrophilz	69.9	81.2	40–70%
Lymphocytes	18.5	12.5	20–45%
Eosinophils	0.7	0.1	<6.0%
Basophils	0.3	0.1	<2.0%
Platelets	366 (10^3^/μL)	179 (10^3^/μL)	140–440 (10^3^/μL)
Creatine serum	1.13	0.55	0.72–1.25 mg/dL
SGOT	15	21	5–34 U/L
SGPT	29	101	<55 U/L
γGT	-	121	10–64 U/L
Ferritine	-	863.52	21.8–274.66 ng/mL
CRP	<0.10	1.64	<0.50 mg/dL
Procalcitonine	0.02	0.05	<0.49 ng/mL
Urine Labs	normal	normal	
HCV	negative	negative	
HbsAG	negative	negative	
Coxiella Burneti IgG	negative	negative	
Coxiella Burneti IgM	negative	negative	
Borrelia burdoferi	negative	negative	
Bartonella quintana IgG	negative	negative	
Bartonella quintana IgM	negative	negative	
Bartonella henselae IgG	negative	negative	
Bartonella henselae IgM	negative	negative	
CSF cultures	negative	negative	
Mantoux test (PPD)	negative		
B. Koch stain and cultures	negative		
Lowenstein-Jensen, Ziehl Neelsen			
ACE	19.2	17.5	8–52 U/L
C3 compl.	116		82–185 mg/dL
C4 compl.	32.7		15–53 mg/dL
ANA	negative		
Anti-Cardiolipine IgG	2.0		<19.9 GPLU/mL
Anti-Cardiolipine IgM	2.6		<12.9 MPLU/mL
Anti-SS-A	negative		
Anti-SS-B	negative		
Anti-Sm	negative		
Anti-RNP	negative		
RF	<5.0		<30 IU/mL
G-6-PDH	14.6		7.0–20.5 U/gHb
pANCA	0.1		9.9 AU/mL
cANCA	0.1		19.9 AU/mL
anti B2-GPI IgG	0.1		19.9 AU/mL
anti B2-GPI IgM	1.6		9.9 AU/mL

Abbreviations: WBC—white blood cells, SGOT—serum glutamic oxalacetic transaminase, SGPT—serum glutamate pyruvate transaminase, γGT—γ-glutamyl transferase, CRP—C-reactive protein, HCV—hepatitis C virus, HbsAG—hepatitis B surface antigen, ACE—angiotensin-converting enzyme, ANA—antinuclear antibodies, Anti-SS-A and -B—anti-Sjögren’s syndrome autoantibodies, Anti-Sm—anti-Sm (Smith) antibodies, Anti-RNP—anti-nuclear Ribonucleoprotein, RF—rheumatoid factor, G-6-PDH—glucose-6-phosphate dehydrogenase, pANCA—perinuclear anti-neutrophil cytoplasmic antibodies, cANCA—cytoplasmic anti-neutrophil cytoplasmic antibodies, anti B2-GPI—antibodies to β2-glycoprotein.

## Data Availability

The data presented in this study are available on request from the corresponding author due to privacy restrictions (containing information that could compromise the privacy of the research participant).

## Abbreviations

The following abbreviations are used in this manuscript:

CT	Computed tomography
CSF	Cerebrospinal fluid
CNS	Central nervous system
MRI	Magnetic resonance imaging
HIV	Human immunodeficiency virus
HBV	Hepatitis B virus
HCV	Hepatitis C virus
PPD	Purified protein derivative
HIT	Heparin induced thrombocytopenia
PCR	Polymerase chain reaction
